# Is the Transrectal Diameter (TRD) Suitable for Assessing Faecal Loads and Monitoring Bowel Management in Children with Hirschsprung Disease—ReKiSo Study: Prospective Study

**DOI:** 10.3390/children11080921

**Published:** 2024-07-30

**Authors:** Judith Lindert, Daniel Erkel, Felix Schulze, Meike Hofer, Edyta Rzepka, Stefanie Märzheuser

**Affiliations:** Department of Paediatric Surgery, University Hospital Rostock, Ernst-Heydemann Str. 8, 18057 Rostock, Germany; daniel.erkel@uni-rostock.de (D.E.); felix.schulze@med.uni-rostock.de (F.S.); meike.hofer@med.uni-rostock.de (M.H.); edyta.rzepka@med.uni-rostock.de (E.R.); stefanie.maerzheuser@med.uni-rostock.de (S.M.)

**Keywords:** Hirschsprung Disease, bowel management, rectal ultrasound, clinician ultrasound

## Abstract

Background: Constipation and outlet obstruction may persist after successful pull-through in Hirschsprung Disease (HD). The radiographic assessment of the faecal load is widely used but exposes the child to radiation. This study aims to evaluate whether the transrectal diameter (TRD) assessed with ultrasound correlates with symptoms of faecal load and whether the TRD normalises when symptoms disappear. Method: Children with HD after pullthrough and functional constipation presenting to our colorectal clinic between 4/23 and 4/24 were assessed for symptoms of constipation, smearing and outlet obstruction, as well as healthy controls. Ultrasound measurement of the TRD was conducted. Bowel management was initiated according to our institutional pathway using Peristeen© irrigation after an orthograde disimpaction regime. Results: A total of 193 children underwent TRD assessment. Of 60 children with HD, 26 (43.3%) presented with obstructive symptoms, and 34 (56.7%) were asymptomatic. In asymptomatic patients with HD, the mean TRD of 2.26 cm (SD 0.61) was significantly (*p* < 0.001) lower than in HD with symptoms, with a mean TRD of 3.35 cm (SD 1.03). Individuals without colorectal pathology had a mean TRD of 2.04 cm (SD 0.37), and children with functional constipation and symptoms showed a mean TRD of 4.36 cm (SD 1.32). The mean TRD after symptom resolution was 2.37 cm. Conclusions: Children with HD without obstructive symptoms have a TRD < 3 cm, as do controls. The transrectal diameter allows the clinician to sonographically assess the faecal load in children with HD at the bedside without radiation. The TRD is useful for monitoring a bowel management program in children with HD.

## 1. Introduction

The long-term follow-up of children with Hirschsprung Disease to assess the development of bowel function is part of the holistic management of Hirschsprung Disease [1,2]. Constipation and outlet obstruction can persist even after technically sound ganglionic pullthrough. Outlet obstruction and constipation are reported in up to 80% of patients with Hirschsprung Disease (HD) [3,4,5]. Several groups report that the length of the aganglionic segment does not determine the probability of persistent symptoms [5]. Children are followed routinely, and bowel management is initiated if necessary. Anatomical causes of post-pullthrough problems need to be excluded during a systematic workup [6,7] and treated, if present. We distinguish children with constipation due to outlet obstruction as a consequence of sphincter achalasia or withholding from children with anatomical causes. Postoperative obstacles such as twisted pullthrough, residual aganglionic segments, large Duhamel’s pouches or other pouches need to be assessed with clinical examination, ultrasound and, if necessary, contrast enema, repeated biopsy and examination under anaesthesia [6,7].

Soiling in children is most likely due to outlet obstruction; damage to the anal canal should also be assessed by clinical examination [6,7]. Recurrent Hirschsprung’s enterocolitis can occur, especially if there are anatomical problems [6,7]. In some children, bowel function problems persist despite a technically sound pullthrough. Defecation disorder is a feature of Hirschsprung’s pathophysiology that needs to be addressed through holistic long-term follow-up. To define faecal impaction, thorough clinical examination and history are essential. To assess the severity, visualisation of the faecal impaction with imaging is crucial.

The radiographic assessment of the stool burden is widely used in both colorectal disease and functional constipation, but it exposes the child to radiation [8,9,10]. Radiographic assessment on plain films is usually made by clinical interpretation [9]. Previously, three different scoring systems have been proposed to assess and evaluate the amount of faecal burden on a plain abdominal radiograph [11]. Assessment on X-ray was first described by Barr in 1979 [12], then by Blethyn in 1995 [13] and then by Leech in 1999 [14]. None of these methods are used routinely in clinical practice. Just recently, a standardised approach to defining different degrees of faecal load and the success of bowel management has been proposed [15]. This approach uses repeated X-ray imaging over several days (5–10) of accumulation of high exposure to radiation [15,16].

Patients with HD require multiple assessments with X-ray during the diagnostic pathway. This exposes the child and, in particular, the pelvic organs such as the ovaries and testes to repeated ionising radiation.

To avoid further radiation exposure, this study aims to measure the transrectal diameter (TRD) to assess the faecal load with ultrasound. Children with HD and children without bowel pathology were investigated. In contrast to previous studies, which excluded children with colorectal disease, we focused on patients with colorectal disease like Hirschsprung Disease [17,18,19]. This study aims to assess whether the transrectal diameter in children with HD correlates with symptoms of stool burden and whether it normalises when symptoms disappear.

## 2. Materials and Methods

**Figure 1:**
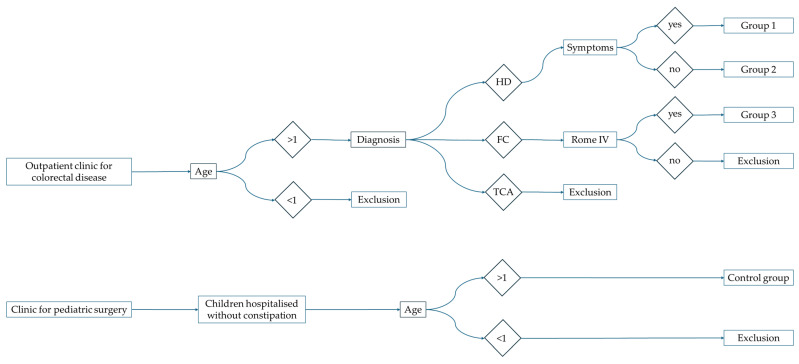
Overview of Patient inclusion and selection.

Children with HD presenting to our colorectal clinic between 4/23 and 4/24 after pullthrough surgery were clinically assessed for symptoms of constipation, stool smearing and outlet obstruction. The treating clinicians routinely performed ultrasound measurements of the TRD (Figure 1). If necessary, bowel management was initiated according to our institutional pathway. Peristeen© irrigation was started after orthograde disimpaction. The required irrigation volume was determined by hydrosonography [20]. If transanal irrigation was indicated, the child was trained to be comfortable with rectal catheter insertion, and each child underwent oral disimpaction just before the hydrosonography.

Families were given the opportunity to familiarise themselves with the irrigation system and were supported by a bowel management nurse and a colorectal case manager. Once the irrigation was started, we routinely followed up with the family after 4 weeks, usually with a telephone follow-up and 3 months later with a regular physical follow-up.

We included children with standard Hirschsprung (*n* = 60) and excluded children with Total Aganglionosis (*n* = 31). We included children with functional constipation (FC, *n* = 51). We included healthy controls without any abdominal pathology (*n* = 51). In this analysis, we excluded other colorectal pathologies such as anal atresia and neurogenic conditions. We excluded children below 1 year of age.

### 2.1. Definition of the Level of Hirschprung Disease

We use the following classification: Type 1—only Rectum, Type 2—Rectosigmoid, Type 3—up to Colon descendens, Type 4—up to Colon ascendens, Type 5—TCA with a maximum of 20 cm of small bowel involvement.

### 2.2. Definition of Symptoms in Children with Hirschsprung Disease

For all children, we assess if their underwear remains clean and if accidental loss appears.

We aim to use the Rintala Bowel Function [1,21] score once the child is toilet-trained for stool to assess bowel function longitudinally.

The Rintala Bowel Function score used to evaluate children with colorectal pathologies assesses the following items: ability to hold back defecation, feels/reports the urge to defecate, frequency of defecation, soiling/staining, accidents/poo in underwear, constipation and social problems.

### 2.3. Definition of Symptoms in Children with Functional Constipation

For functional constipation, we additionally use the Rome IV criteria [22]. Stool consistency was reported according to Bristol Stool chart.

### 2.4. Transrectal Ultrasound Assessment

**Figure 2:**
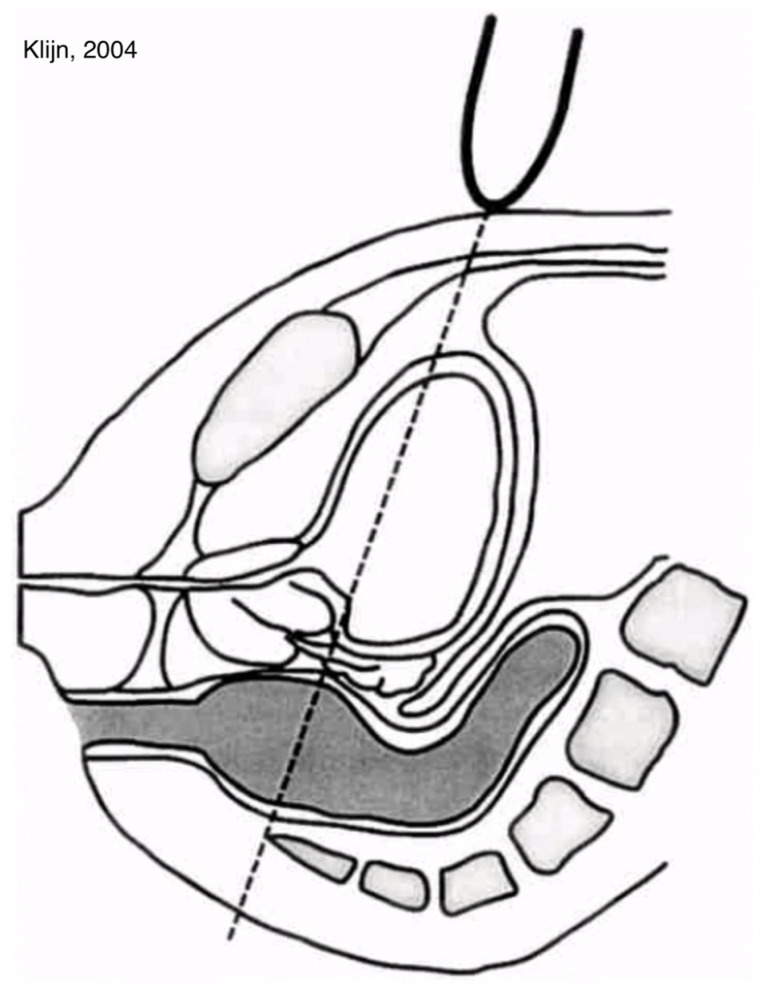
Schematic Graphic; TRD according to Klijn. Reprinted with permission from [23].

We used the method of Klijn [23] by placing a curved array of 3.5 MHz (Toshiba Aplio 300, Toshiba Medical Systems GmbH, Neuss, Germany) above the symphysis and measured the largest TRD at a downward angle of 15 degrees from the transverse plane; see Figure 2.

**Figure 3:**
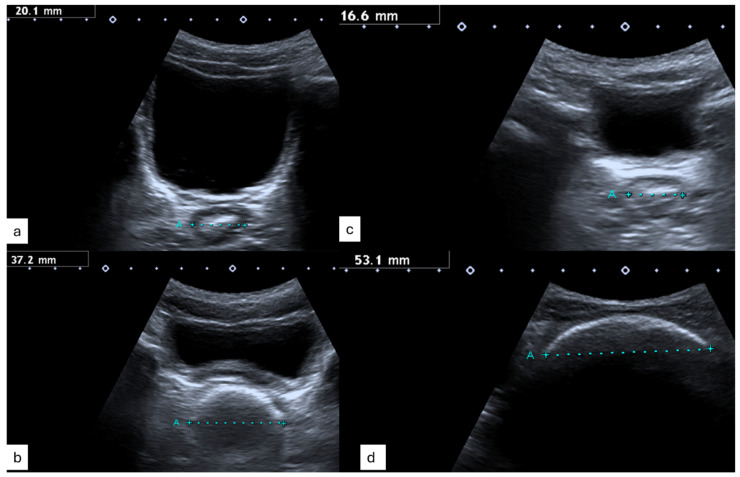
Transrectal Diameter and Picture Constipation of (**a**) a child with HD and normal bowel function, (**b**) a child with HD and obstructive symptoms, (**c**) a healthy control and (**d**) a child with functional constipation and symptoms.

Figure 3 displays typical ultrasound images with a normal TRD (Figure 3a,c) and feacal load (Figure 3b,d) in children with Hirschsprung Disease (Figure 3a,b), healthy controls (Figure 3c) and those with functional constipation (Figure 3d).

## 3. Results

We included 162 children with sonographic assessment of the transrectal diameter. There were a total of 60 children with standard Hirschsprung Disease, of whom 26 (43.3%) presented with outlet obstruction/constipation symptoms and 34 (56.7%) presented without any symptoms. We scanned 51 controls without any symptoms and 51 children with functional constipation.

In this cohort we have follow-up data with a mean of 44.3 months after pullthrough for the children with HD (76% males, 24% females). In total, 34 (56.7%) children had no obstructive symptoms, and 26 (43.3%) had symptoms at the index assessment. The patient characteristics are shown in Table 1.

**Figure 4:**
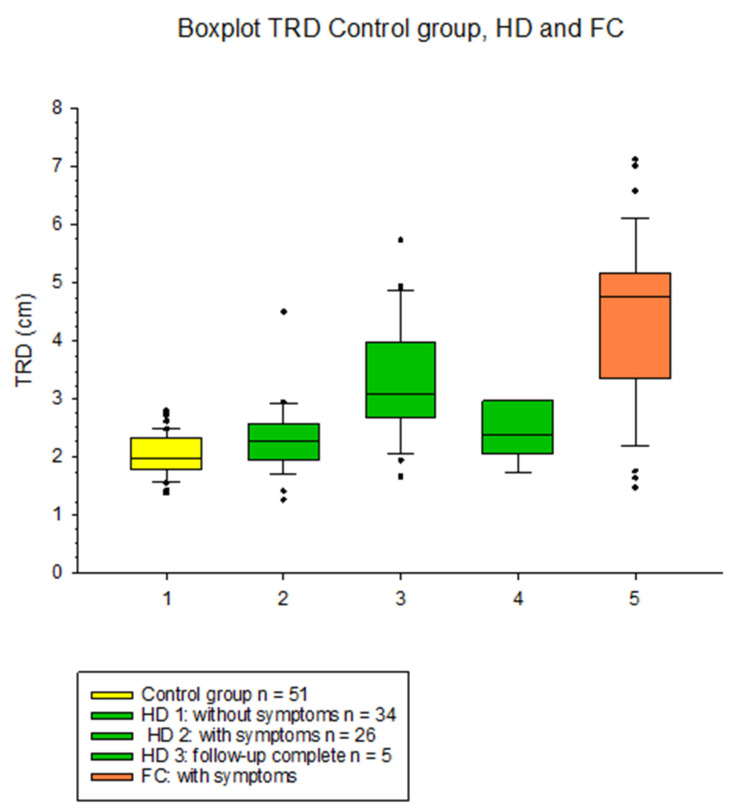
Boxplots TRD in control children (yellow), children with Hirschsprung without outlet obstruction/constipation (HD1), children with HD and outlet obstruction/constipation (HD2) and with successful bowel management (HD3) and children with functional constipation with symptoms (orange).

A total of 162 children underwent a sonographic evaluation of TRD. A total of 60 children had HD, of whom 26 (43.3%) had symptoms of outlet obstruction/constipation and 34 (56.7%) had no symptoms. We scanned 51 controls without symptoms and 51 children with functional constipation. In asymptomatic patients with HD, the mean TRD of 2.26 cm (SD 0.61) was significantly (*p* < 0.001) lower than in HD with symptoms, with a mean TRD of 3.35 cm (SD 1.03); see Figure 4.

Individuals (*N* = 34) without colorectal pathology had a mean TRD of 2.04 (SD 0.37), and children with functional constipation and symptoms had a mean TRD of 4.36 cm (SD 1.32). The TRD was significantly larger when children presented with symptoms regardless of the cause; interestingly, children with functional constipation presented with a significantly (*p* = 0.001) larger TRD when comparing HD with symptoms and FC.

Age-appropriate bowel management was initiated using transanal irrigation with a Foley catheter or Peristeen©. The mean time to the resolution of obstructive symptoms was 3.25 months, and the mean TRD after the resolution of symptoms was 2.37 cm.

The irrigation volume was individually determined by hydrosonography and varied between 200 and 800 mL, highlighting the importance of individual ultrasound assessment in each case.

## 4. Discussion

We report on the prospective evaluation of 60 children with standard Hirschsprung Disease at a mean of 44.3 months after pullthrough compared to 51 children with functional constipation in our colorectal clinic. The sonographic transrectal diameter (TRD) was compared with that of 51 healthy controls. In our cohort, 26 children with HD (43.3%) presented with outlet obstruction/constipation and a mean transrectal diameter of 3.35 cm with symptoms. The main symptom was soiling in 54% and constipation in 46% of children with symptoms. When symptoms were resolved with tailored bowel management, these children had a significantly (*p* = 0.001) smaller mean TRD of 2.49 cm.

We performed routine transrectal ultrasound assessments in all children with Hirschsprung Disease as well as controls without any colorectal pathology [24].

A cut-off value of 3 cm in diameter has been validated for children without colorectal pathology to differentiate between constipation and regular defecation [17,18,19].

In our patients, we have confirmed that this cut-off reliably distinguishes (HD with symptoms vs. without symptoms: *p* < 0.001) patients with Hirschsprung Disease and impaired bowel function from symptom-free HD children.

The pulled through bowel takes over the function of the resected rectum. A filling greater than 3 cm indicates an increased faecal load, which is often associated with impaired bowel function.

### 4.1. Bowel Management in Hirschsprung

With our individualised yet standardised bowel management program, the transrectal diameter on ultrasound diminished and was lower (⌀2.49 cm) than the proposed cut-off when symptoms disappeared at a mean follow-up of 3.25 months.

The bowel management is initiated with ultrasound control. For toddlers, we opt for passive irrigation using a Silicon Foley and syringe and teach the parents to perform those washouts.

Usually around 3–4 years, we introduce active irrigation with Peristeen© Special to our practice is that we determine the amount of irrigation fluid using ultrasound. Other groups empirically judge the amount or would use a contrast enema [15].

This bedside ultrasound by us enables the families and child to directly observe the irrigation process and engages the patient actively.

Likewise, other groups note that the understanding and cooperation of the patient and parents is crucial for the success of transanal irrigation regardless of the underlying cause [20,25,26].

We empower children to participate early and note that from around 8 years, many can conduct their irrigations independently [27].

Other groups routinely use Botox when outlet obstruction persists, which relieves the Sphincter Achalasia. The effect usually is present for 3–6 months, and the administration may be repeated, but anaesthesia is needed for the intrasphincterial administration. However, transanal irrigation supplements the effect in Botox and should ideally be performed at the same time.

### 4.2. Bowel Management in Chronic Constipation

Our data on 51 children with functional constipation showed that the sonographic monitoring of TRD supports the implementation of a tailored bowel management programme. The main presenting symptoms were constipation (94%) and soiling (65%). With a success rate of 90% with transanal irrigation (either Peristeen© or Quofora Irrisedeo Mini Go^®^), we note a higher likelihood of becoming clean compared to others describing the antegrade continence enema (ACE) route, with a success rate of 31.5% [28]. Children are clean on clinical symptoms, and the TRD decreases significantly from abnormal (⌀4.36 cm) to normal (⌀2.48 cm) when symptoms disappear.

Supervised training and sustained follow-up are key features to achieving the best possible outcome with irrigation in children with functional or organic bowel dysfunction [25]. In both Hirschsprung Disease, due to persistent sphincter achalasia caused by impaired anorectal inhibitory reflex, and functional constipation, usually associated with some degree of withholding behaviour, it may be particularly important to use transanal irrigation rather than antegrade irrigation to overcome the outlet obstruction by direct stimulation [29]. Over time, families and especially patients benefit greatly from learning self-management strategies and becoming independent in their bowel management. Self-management has been described as the key element in multidisciplinary disease management strategies in chronic disease care [27].

## 5. Conclusions

This prospective longitudinal assessment of the transrectal diameter on clinician ultrasounds in children with Hirschsprung and controls without colorectal pathology enables the detailed measurement and definition of a transrectal diameter in children with Hirschsprung Disease for the first time.

Children with HD without obstructive symptoms have a transrectal diameter < 3 cm, as do controls. The transrectal diameter allows the clinician quickly to sonographically assess the faecal load in children with HD at the bedside without radiation. The TRD is useful for monitoring a bowel management program in children with HD.

## Figures and Tables

**Table 1:** Patient characteristics.

	Hirschsprung with Symptoms *N* = 26	Hirschsprung without Symptoms *N* = 34	Control without Symptoms *N* = 51	Functional Constipation *N* = 51	*p*-Value
Gender (female, male)	7:19 (27%:73%)	6:28 (18%:72%)	36:15 (71%:29%)	26:25 (51%:49%)	
Age at pullthrough (months, mean)	13	23	-	-	
Age at rectal ultrasound (years, mean)	4.85	5.23	7	7	- HD with vs. without symptoms: *p* = 0.457 - HD with symptoms vs. Control: *p* = 0.032 - HD with symptoms vs. FC: *p* = 0.067
Months after pullthrough (mean, min.–max.)	45.2 (8–161)	38.42 (0–191)	-	-	
Mean transrectal diameter TRD at first scan (cm)	3.45, SD 1.01	2.31, SD 0.57	2.05, SD 0.37	4.36, SD 1.39	- TRD HD with symptoms vs. without symptoms: *p* < 0.001 - TRD HD without symptoms vs. CG: *p* = 0.02 - TRD HD with symptoms vs. FC: *p* = 0.001 - TRD HD follow-up vs. FC follow-up: *p* = 0.874
Symptoms Yes/No	Yes	No	-	Yes 26 (51%) No 34 (49%)	
Symptoms - soiling - stool accidents - distended abdomen	- soiling: 14 (54%) - distended abdomen: 6 (23%) - constipation: 12 (46%)	-	-	- soiling: 17 (65%) - distended abdomen: 7 (27%) - constipation (94%) - faecaloma: 15 (58%)	
Months last follow-up (mean)	4.6		-	3.3	
Transrectal diameter TRD last follow-up (mean)	2.49, SD 0.5			2.48, SD 0.36	

## Data Availability

The data presented in this study are available on request from the corresponding author. The data are recorded in an anonymous institutional database due to privacy or ethical restrictions.

## Abbreviations

HD	Hirschsprung Disease
TRD	Transrectal diameter
FC	Functional constipation
ACE	Antegrade Continence Enema
